# Systems for Rapidly Detecting and Treating Persons with Ebola Virus Disease — United States

**Published:** 2015-03-06

**Authors:** Lisa M. Koonin, Denise J. Jamieson, John A. Jernigan, Chris A. Van Beneden, Christine Kosmos, Melissa Cole Harvey, Harald Pietz, Jeanne Bertolli, Joseph F. Perz, Cynthia G. Whitney, Alison Sheehan-Laufer Halpin, W. Randolph Daley, Nicki Pesik, Gregg S. Margolis, Abbigail Tumpey, Jordan Tappero, Inger Damon

**Affiliations:** 1 Influenza Coordination Unit, Office of Infectious Diseases, CDC; 2 Division of Reproductive Health, National Center for Chronic Disease Prevention and Health Promotion, CDC; 3 Division of Healthcare Quality Promotion, National Center for Emerging and Zoonotic Infectious Diseases, CDC; 4 Division of Bacterial Diseases, National Center for Immunization and Respiratory Diseases, CDC; 5 Division of State and Local Readiness, Office of Public Health Preparedness and Response, CDC; 6 Division of National Healthcare Preparedness Programs, Office of the Assistant Secretary for Preparedness and Response, US Department of Health and Human Services; 7 Division of Public Health Performance Improvement, Office for State, Tribal, Local, and Territorial Support, CDC; 8 Division of HIV/AIDS Prevention, Surveillance and Epidemiology, National Center for HIV/AIDS, Viral Hepatitis, STD, and TB Prevention, CDC; 9 Office of the Director, National Center for Emerging and Zoonotic Infectious Diseases, CDC; 10 Division of Health Systems Policy, Office of the Assistant Secretary for Preparedness and Response, US Department of Health and Human Services; 11 Division of Global Health Protection, Center for Global Health, CDC; 12 Division of High-Consequence Pathogens and Pathology, National Center for Emerging and Zoonotic Infectious Diseases, CDC

The U.S. Department of Health and Human Services (HHS), CDC, other U.S. government agencies, the World Health Organization (WHO), and international partners are taking multiple steps to respond to the current Ebola virus disease (Ebola) outbreak in West Africa to reduce its toll there and to reduce the chances of international spread. At the same time, CDC and HHS are working to ensure that persons who have a risk factor for exposure to Ebola and who develop symptoms while in the United States are rapidly identified and isolated, and safely receive treatment. HHS and CDC have actively worked with state and local public health authorities and other partners to accelerate health care preparedness to care for persons under investigation (PUI) for Ebola or with confirmed Ebola. This report describes some of these efforts and their impact.

## Traveler Screening

Since the beginning of August 2014, CDC, WHO, and other global partners have assisted the ministries of health of the three countries with widespread transmission (Guinea, Liberia, and Sierra Leone) and Mali (during November 17, 2014–January 5, 2015) to develop and implement exit screening intended to reduce the likelihood of international spread of Ebola (1). Exit screening procedures include administering a health questionnaire, measuring body temperature, and, if there is a fever, conducting an assessment of the likelihood of the fever being caused by Ebola. Travelers who have fever or symptoms compatible with Ebola or who report a high risk for exposure to Ebola are denied boarding on international flights (1). In addition, enhanced screening of all travelers arriving in the United States from the three countries with widespread transmission is being conducted (i.e., entry screening) (1). This program allows federal authorities to assess arriving travelers and link them with state and local partners to facilitate ongoing health monitoring and prompt referral to care if appropriate. Currently, all travelers from Guinea, Liberia, and Sierra Leone are routed to arrive in five designated U.S. international airports (New York’s John F. Kennedy, Washington-Dulles, Newark’s Liberty, Chicago’s O’Hare, and Atlanta’s Hartsfield-Jackson airports), where they undergo enhanced screening upon arrival (1). Any of these travelers who have a possible risk for having been exposed to Ebola virus are referred to CDC public health officers stationed at the airport for a more detailed risk assessment. Travelers who have fever or other symptoms compatible with Ebola are also promptly referred to CDC on-site for further evaluation and subsequently for medical evaluation and care at a local hospital if needed. During October 11, 2014–January 31, 2015, a total of 7,587 persons arriving from affected countries* have been screened upon entry to the United States. Of these, 543 (7.2%) were referred to on-site CDC screening at the airport for additional exposure risk assessment. At the time of assessment, 12 (0.16%) travelers were referred for medical evaluation at a local hospital, and none had Ebola diagnosed.

CDC notifies state and local public health officials within hours of entry into the United States of all arriving travelers entering their jurisdiction from the affected countries who require monitoring (2). Public health departments then monitor these travelers until 21 days have elapsed since their departure from an Ebola-affected country (2). Travelers are required to measure their temperature a minimum of twice per day and monitor themselves daily for other symptoms of Ebola. Certain travelers (and others identified by public health authorities) at greater risk for exposure (e.g., persons who had provided health care to a patient with Ebola) are required to report twice daily to public health authorities; one daily report must include direct visual contact (2). This active monitoring effort aims to rapidly identify any recently arrived traveler who develops signs and symptoms compatible with Ebola so they can be appropriately referred for medical evaluation and diagnosis.

Any person with an epidemiologic risk factor† within the preceding 21 days who develops symptoms compatible with Ebola is considered a PUI.§ During October 11, 2014–January 31, 2015, at least 136 persons were identified as PUIs and were referred for evaluation and treatment. None of these PUIs had Ebola; the most common diagnoses were malaria and influenza (3).

## U.S. Hospital and Health Care Facility Preparedness: A Tiered Approach

Active monitoring by public health officials aims to identify persons who are at risk for Ebola and might be developing early symptoms of Ebola so they can be isolated and receive immediate evaluation and care. Rapid and careful treatment of persons with confirmed Ebola by appropriately trained health care personnel reduces the possibility of secondary transmission and might lead to improved outcomes. Although Ebola infections in the United States are extremely rare, the disease is typically very severe and can present a risk for transmission in health care settings, particularly in the later stages of illness. Management of infected persons requires dedicated facilities, highly trained staff, and use of recommended personal protective equipment (PPE) (4,5).

CDC and the Office of the Assistant Secretary for Preparedness and Response (ASPR) at HHS have developed a tiered approach to prepare U.S. health care facilities to safely and rapidly identify, isolate, evaluate, manage, and transfer (if needed) travelers or patients who have possible or confirmed Ebola (6). To ensure that a network of prepared facilities is available to serve in this capacity, CDC and ASPR, in collaboration with state and local public health authorities, rapidly provided technical assistance to hospitals that are strategically located near airports with a large number of travelers returning from Ebola-affected countries and in communities where large numbers of persons from these West African countries reside.

Acute health care facilities serve one of three roles: frontline health care facilities, Ebola assessment hospitals, and Ebola treatment centers. Some hospitals serve simultaneously as Ebola assessment hospitals and Ebola treatment centers. CDC and ASPR’s framework for this national approach includes:

- **Frontline health care facilities.** Most U.S. acute care facilities that are equipped for emergency care (e.g., hospital-based emergency departments and other emergency care settings, including urgent care clinics) are in this tier. Because PUIs will be directed to other designated facilities and travelers returning from West Africa are being screened and monitored, patients with unrecognized Ebola are unlikely to present to frontline health care facilities without notification of their arrival, although the possibility exists. However, patients might be temporarily referred to a frontline health care facility when it is not feasible to refer to other designated facilities based on distance, bed availability, or other considerations. Therefore, frontline health care facilities should be prepared to rapidly identify and isolate patients who might have Ebola and promptly inform the hospital/facility infection control program and state and local public health agencies (7). Frontline health care facilities will quickly transfer these patients to an Ebola assessment hospital or Ebola treatment center as recommended by state and local public health authorities.
- **Ebola assessment hospitals.** These facilities are prepared to receive and isolate a PUI and care for the patient until a diagnosis of Ebola can be confirmed or ruled out and until discharge or transfer is completed, which can take up to 96 hours (8). Ebola assessment hospitals should also be equipped to effectively evaluate and treat other conditions (e.g., malaria and influenza) using appropriate diagnostics and therapies. Patients with confirmed Ebola should be transferred to an Ebola treatment center, according to the state’s plan. Nearly every state has identified at least one Ebola assessment hospital.
- **Ebola treatment centers.** These hospitals are prepared to provide comprehensive care to persons diagnosed with Ebola for the duration of a patient’s illness (9). State and local health officials, in consultation with hospital leadership and CDC, have currently designated Ebola treatment centers and have conducted extensive preparedness activities. As of February 18, 2015, there were 55 U.S. hospitals with Ebola treatment centers (10). Most Ebola treatment centers have agreed to serve as a resource for their state; a smaller number are likely to be willing to care for patients from outside their state or outside the United States. The three U.S. biocontainment units (Emory University Hospital, the National Institutes of Health Clinical Center, and Nebraska Medicine) also serve as Ebola treatment centers.

## Preparing Ebola Treatment Centers in the United States

Although all health care facilities should be able to quickly identify a patient with a history or symptoms consistent with Ebola, limited numbers are needed to further assess a patient for Ebola or manage an Ebola patient for the course of their illness. Designating a facility as an Ebola treatment center has been a collaborative decision made jointly by state and local health authorities and the hospital administration. These decisions have been informed by the results of CDC site visits conducted by interdisciplinary teams of subject matter experts. CDC assembled Rapid Ebola Preparedness (REP) teams that have visited more than 80 hospitals in 20 states and the District of Columbia. REP teams have assessed facilities’ infection control readiness at the request of local and state health authorities; however, public health officials and the health systems within their jurisdictions identified the hospitals that are best suited to safely care for PUIs or patients with confirmed Ebola and are ultimately responsible for designating Ebola treatment centers. REP teams usually consisted of 4–10 persons, with a CDC employee serving as the lead and included CDC staff members, ASPR staff, professional partners from the Association for Professionals in Infection Control and Epidemiology, the Infectious Diseases Society of America, and the Society for Healthcare Epidemiology of America, experts in clinical care from the three U.S. biocontainment units with experience treating Ebola patients, and additional federal partners (e.g., the U.S. Department of Veterans Affairs and the U.S. Department of Labor’s Occupational Safety and Health Administration).

REP teams offered technical assistance and guidance, including recommendations for additional training and technical support. During the visits, REP teams helped hospitals identify gaps in their Ebola-specific infection control plans; focal areas included infection control practices, worker safety, diagnostics, laboratory processes, waste management, and other key areas (9). Follow-up and technical support was provided to these hospitals to facilitate implementation of REP team recommendations.

## Next Steps

Many Ebola treatment centers have been designated, particularly in the geographic regions that have the largest numbers of West African travelers and expatriates. To ensure effective evaluation and treatment of PUIs, increased focus is being placed on the identification and evaluation of Ebola assessment hospitals. Because persons might require care anywhere in the United States, public health authorities will be promptly directing those persons to Ebola assessment hospitals for evaluation as soon as they report one or more symptoms. These assessment facilities must be available and prepared to evaluate a patient with little advance notice, if necessary.

In addition, experts in caring for Ebola patients and stakeholder groups have suggested that, to the extent possible, care of patients with Ebola should be concentrated in a small number of well-prepared facilities. Therefore, building upon the state-based and jurisdiction-based tiered hospital approach, ASPR is developing a regional approach to caring for future patients with Ebola, in which up to 10 Ebola treatment centers will be designated to serve as regional Ebola and other special pathogens treatment centers (one in each of the 10 HHS regions).¶ These regional centers will have enhanced capacity and capabilities to care for patients with Ebola and other highly infectious diseases, and they will be ready within a few hours to receive a patient with confirmed illness from their region or from across the United States, or a patient who has been medically evacuated from outside of the United States. Patients with confirmed Ebola will be preferentially referred to one of these regional centers, as necessary. Finally, CDC Ebola response teams are deployed by request, to any Ebola treatment center or hospital with a confirmed or highly suspected case of Ebola to provide technical assistance for infection control procedures, clinical care, and logistics of managing a patient with Ebola.

## Conclusion

As the Ebola outbreak in West Africa continues, the United States will need to maintain capabilities to detect and manage persons with possible or confirmed Ebola. Current efforts to improve health care facility readiness for Ebola will continue to be responsive to the current situation in West Africa and will continue to evolve as the situation changes. The efforts and infrastructure being developed to rapidly identify, evaluate, and treat persons with possible Ebola in the United States will likely improve the outcomes of these patients, reduce the spread of Ebola to others, and also help prepare for future emerging infectious disease threats.

## Footnotes

* Additional information on countries affected by Ebola outbreaks is available at http://www.cdc.gov/vhf/ebola/outbreaks/2014-west-africa/distribution-map.html#areas.

† Additional information on epidemiologic risk factors for Ebola is available at http://www.cdc.gov/vhf/ebola/exposure/risk-factors-when-evaluating-person-for-exposure.html.

§ A person who has signs or symptoms consistent with Ebola (i.e., elevated body temperature or subjective fever or symptoms [including severe headache, fatigue, muscle pain, vomiting, diarrhea, abdominal pain, or unexplained hemorrhage] and an epidemiologic risk factor within the 21 days before the onset of symptoms) should be considered a PUI.

¶ Additional information available at http://www.grants.gov/web/grants/view-opportunity.html?oppId=274709.
